# Evaluating distributed-learning on real-world obstetrics data: comparing distributed, centralized and local models

**DOI:** 10.1038/s41598-024-61371-1

**Published:** 2024-05-15

**Authors:** João Coutinho-Almeida, Ricardo João Cruz-Correia, Pedro Pereira Rodrigues

**Affiliations:** 1 CINTESIS@RISE-Centre for Health Technologies and Services Research, University of Porto, Porto, Portugal; 2 MEDCIDS-Faculty of Medicine, University of Porto, Porto, Portugal; 3 Health Data Science PhD Program, Faculty of Medicine, University of Porto, Porto, Portugal

**Keywords:** Computer science, Data processing, Machine learning, Statistical methods

## Abstract

This study focused on comparing distributed learning models with centralized and local models, assessing their efficacy in predicting specific delivery and patient-related outcomes in obstetrics using real-world data. The predictions focus on key moments in the obstetric care process, including discharge and various stages of hospitalization. Our analysis: using 6 different machine learning methods like Decision Trees, Bayesian methods, Stochastic Gradient Descent, K-nearest neighbors, AdaBoost, and Multi-layer Perceptron and 19 different variables with various distributions and types, revealed that distributed models were at least equal, and often superior, to centralized versions and local versions. We also describe thoroughly the preprocessing stage in order to help others implement this method in real-world scenarios. The preprocessing steps included cleaning and harmonizing missing values, handling missing data and encoding categorical variables with multisite logic. Even though the type of machine learning model and the distribution of the outcome variable can impact the result, we reached results of 66% being superior to the centralized and local counterpart and 77% being better than the centralized with AdaBoost. Our experiments also shed light in the preprocessing steps required to implement distributed models in a real-world scenario. Our results advocate for distributed learning as a promising tool for applying machine learning in clinical settings, particularly when privacy and data security are paramount, thus offering a robust solution for privacy-concerned clinical applications.

## Introduction

As the use of Artificial Intelligence (AI) is increasing in the healthcare space^1^, increased demand for ethical usage of personal patient data is occurring as well^2^. This has been happening both on the governmental side, with several regulations passed to protect citizens’ data and personal information (such as GDPR in the EU^3^ and HIPPA in the US^4^), and on the public side, with an increased concern with continuous data breaches across institutions^5^. So, we are now faced with a dilemma on a compromise between what is possible to do with the available data and what should be done regarding patient privacy^6^. This is the main reason why health institutions implement burdensome processes and methodologies for sharing patient data, often costing a great deal of time, money, and human resources, seldomly overtaking the ideal time frame for analysing such data. Due to these privacy concerns, the traditional method for using data in healthcare is, nowadays, by focusing on data from a single institution in order to predict or infer something regarding those patients; this could be understood as local learning. This approach has some drawbacks, namely data quantity, data quality and possible class imbalance^7^, never quite raising into its full potential for promoting best healthcare practices^8–10^ with data sharing between institutions. In order to overcome this issue, there are a few, more complex, systems that consolidate data from several institutions, so more robust algorithms could be trained. However, this globally centralised consolidation of data encompasses a very important data breach hazard.

This is the setting where distributed learning could create a greater impact. A halfway point between local and centralised learning is where we train several models, one in each institution (or silo), and where the sole information that leaves the premises is a trained model or its metadata. A distributed model is built as the aggregation of all the local models, consequently aiming to create a model similar to one globally trained with all the data in a centralised server. However, the distributed model never contacted with any data, only the local models did. This provides the opportunity to create better models, improve data protection, reduce training time and cost and provide better scaling capabilities^11^.

While numerous multi-institutional initiatives have successfully established integrated data repositories for healthcare research, there remains an incomplete understanding of the performance and scalability of distributed systems when directly compared to traditional, centralised models. Specifically, the nuanced behaviors of these distributed frameworks under real-world data conditions-contrasted against classical models that utilize consolidated data-have yet to be fully delineated. This paper aims to critically evaluate the efficacy and suitability of distributed mechanisms within the healthcare domain, assessing their potential as viable alternatives to conventional machine-learning pipelines. The objectives of this paper include:

- Evaluate a distributed model against its local counterparts and against the centralized version;
- Describe the preprocessing required to implement distributed learning with real world data;

## Theoretical background and related work

Distributed learning^12^ can be understood as training several models in a different setting and then aggregating them as a whole. There are two main branches of these approaches, distinguishable by the existence of a central orchestrator server: federated learning where such an entity exists, and peer-to-peer (or swarm)^6^ learning where it does not. Distributed learning can be implemented in various ways, depending on the chosen base algorithm. One common method is averaging the weights of models, primarily utilized in federated averaging. Alternatively, distributed systems can aggregate individual models into an ensemble, enhancing performance by leveraging the strengths of different models^12^. Even though distributed learning has been receiving a lot of attention recently, only some of its concepts have been focused on, mainly distributed-deep learning with a federated learning approach^13,14^. These methods use the strength of neural networks and several algorithms such as federated averaging to create distributed models capable of handling complex data like text, sound, or image^15^. However, considering that there are great amounts of information, especially in healthcare, stored as tabular data^16–20^ and that neural networks are often not the best tool for such data structures and often outperformed by boosting algorithms and tree based models^21,22^, there is a lack of knowledge in the traditional machine learning techniques in a distributed manner. This is specially important since tabular data comes mainly from Electronic Health Records (EHRs) and this kind of data is often of lower quality, with missing values, and with a high number of categorical variables and unstructured/semi-structured variables which make the application of classical machine-learning algorithms harder than for example images, which are mainly computer and systematically generated^23^.

Nevertheless, there have been some health-related distributed machine-learning projects successfully implemented, such as euroCAT^24^ which implemented an infrastructure across five clinics in three countries. SVM models were used to learn from the data distributed across the five clinics. Each clinic has a connector to the outside where only the model’s parameters are passed to the central server which acts as a master deployer regarding the model training with the radiation oncology data. Also, ukCAT^25^ did similar work, with an added centralised database in the middle, but the training being done with a decentralized system. There are also reports of a study that introduces “confederated machine learning” for modeling health insurance data that is fragmented both horizontally (by individual) and vertically (by data type), without the need for central data consolidation. It showcases the method’s efficacy in predicting diseases like diabetes and heart conditions across data silos, achieving notable prediction accuracy, thereby advancing federated learning in healthcare by accommodating complex data separations and enhancing model training without compromising patient privacy or data security^26^. Distributed initiatives have also been covered in a review by Kirienko et al.^27^, where we can see very few papers have described a distributed learning approach without federation. However, from these, we can highlight the works of Wang et al.^28^ tried to use these approaches to detect re-hospitalization for heart failure and Tuladhar et al.^12^ where they used the distributed approach to detect several diseases like diabetes, heart disease, and mild cognitive impairment.

Several studies have examined model evaluation in distributed settings, such as comparing centralized and distributed machine learning using the MNIST dataset^29^. Others have evaluated federated learning on the MNIST, MIMIC-III, and PhysioNet ECG datasets, though these studies did not compare federated learning to other methodologies^30^. Tuladhar and colleagues have also investigated healthcare images and various public and curated datasets^12^. Morevoer, none of them address the challenges of using real-world data and how to implement these methodologies in live scenarios. Additionally, a scoping review emphasizes the importance of thoroughly evaluating distributed and federated models against local models^31^. To our knowledge, this study is the first to evaluate distributed machine learning on such a broad scale with real-world tabular clinical data from nine distinct sources, employing various algorithms and outcome variables, and comparing these methods to both centralized and local approaches. This evaluation encompasses both federated and peer-to-peer methodologies.

## Materials

Clinical data was gathered from nine different Portuguese hospitals regarding obstetric information, pertaining to admissions from 2019 to 2020. This originated nine different files representing different sets of patients but with the same features associated to them. The software for collecting data was the same in every institution (although different versions existed across hospitals) - ObsCare^32^. The data columns are the same in every hospital’s database. Each hospital was considered a silo and summary statistics of the different silos are reported in the Tables 1 and 2. The data dictionary is in Appendix A. The datasets were anonymized and de-identified prior to analysis and each hospital was assigned a number to ensure confidentiality. Each dataset represents a different hospital, which we will use for this analysis as a isolated silo and the number of patients in each dataset is reported in the last row of the Tables 1 and 2. Dataset comprised of patient’s features like age and weight and characteristics as well, like if the patient smoked during pregnancy or had gestational diabetes. The dataset also comprises information about the pregnancy like number of weeks, type of birth, bishop score (pre-labor scoring system used to predict the success of induction of labor), or if the pregnancy was followed by a specific physician in a specific scenario.

This study received Institutional Review Board approval from all hospitals included in this study with the following references: Centro Hospitalar São João; 08/2021, Centro Hospitalar Baixo Vouga; 12-03-2021, Unidade Local de Saúde de Matosinho; 39/CES/JAS, Hospital da Senhora da Oliveira; 85/2020, Centro Hospitalar Tamega Sousa; 43/2020, Centro Hospitalar Vila Nova de Gaia/Espinho; 192/2020, Centro Hospitalar entre Douro e Vouga; CA-371/2020-0t_MP/CC, Unidade Local de saúde do Alto Minho; 11/2021. All methods were carried out in accordance with relevant guidelines and regulations.

**Table 1:** Silos overview.

Variable	Silo 1	Silo 2	Silo 3	Silo 4	Silo 5	Total
N (total)	8039	8566	4989	2364	18177	80874
**Actual type of delivery C (%)**	10 (52.6)	3 (51.6)	3 (57.8)	3 (61.8)	9 (61.5)	11 (52.9)
Bishop Score C (%)	15 (98.5)	15 (78.8)	13 (97.4)	16 (86.4)	15 (97.4)	16 (95.3)
**Blood group C (%)**	9 (39.9)	10 (39.9)	9 (39.3)	11 (37.9)	10 (40.9)	14 (40.5)
**Body mass index** $\mu (\sigma )$	25.2 (8.6)	25.2 (6.2)	25.0 (5.3)	25.0 (8.9)	24.9 (7.8)	25.1 (7.0)
Cervical consistency C (%)	4 (98.6)	4 (83.4)	4 (99.3)	4 (87.4)	4 (97.5)	4 (96.5)
Cervical position C (%)	4 (98.6)	4 (83.3)	4 (99.3)	4 (87.5)	4 (97.6)	4 (96.6)
**Delivery type C (%)**	6 (43.4)	6 (53.5)	5 (44.4)	7 (52.2)	7 (49.3)	8 (51.3)
Dilatation C (%)	5 (98.5)	5 (83.1)	5 (99.3)	5 (87.2)	5 (97.5)	5 (96.5)
Effacement C (%)	5 (98.6)	5 (83.2)	5 (99.3)	5 (87.2)	5 (97.5)	5 (96.5)
Fetal station C (%)	5 (98.6)	5 (83.3)	5 (99.3)	5 (87.9)	5 (97.5)	5 (96.6)
**Followed physician C (%)**	3 (99.2)	4 (92.2)	3 (99.1)	3 (94.3)	3 (99.0)	4 (97.9)
**Followed physician hospital delivery C (%)**	2 (87.6)	2 (75.8)	2 (81.4)	2 (52.2)	2 (71.0)	2 (69.0)
**Followed physician primary care C (%)**	2 (61.3)	2 (52.8)	2 (78.1)	2 (50.4)	2 (70.4)	2 (67.6)
Followed physician private clinic C (%)	2 (81.8)	2 (85.0)	2 (80.6)	2 (78.8)	2 (73.3)	2 (75.8)
Gestational diabetes C (%)	2 (87.7)	2 (90.0)	2 (90.2)	2 (90.8)	2 (89.8)	2 (89.5)
Induced delivery C (%)	2 (97.8)	2 (83.9)	2 (93.3)	2 (91.9)	2 (98.5)	2 (92.5)
**Mother age** $\mu (\sigma )$	31.1 (5.7)	30.7 (5.6)	31.1 (5.9)	31.1 (6.3)	31.3 (5.6)	31.1 (5.6)
Nr Deliveries forceps C (%)	4 (99.2)	3 (83.3)	4 (94.3)	4 (95.8)	3 (60.1)	5 (82.6)
Nr Deliveries no assistance C (%)	10 (74.7)	9 (60.3)	9 (74.9)	9 (67.3)	11 (45.4)	12 (60.3)
Nr Deliveries vacuum C (%)	5 (90.4)	4 (79.9)	4 (89.0)	4 (93.1)	5 (55.3)	5 (77.4)
Nr of C-sections C (%)	6 (87.9)	6 (72.6)	5 (86.1)	5 (89.5)	6 (62.1)	6 (74.6)
**Nr of pregnancies C (%)**	11 (40.9)	11 (43.1)	13 (39.1)	12 (38.7)	16 (42.8)	19 (42.1)
**Nr of born babies C (%)**	10 (44.8)	10 (41.4)	10 (36.9)	10 (42.0)	12 (35.3)	12 (38.8)
**Nr of consultations** $\mu (\sigma )$	7.3 (4.7)	7.0 (6.4)	6.4 (3.9)	5.5 (3.6)	10.5 (5.1)	8.4 (5.1)
Pelvis Adequacy C (%)	4 (95.4)	4 (77.7)	4 (90.1)	3 (96.9)	4 (81.2)	4 (82.6)
**Position admission C (%)**	5 (88.5)	6 (78.0)	6 (51.8)	3 (95.9)	6 (71.3)	7 (73.1)
**Position on delivery C (%)**	5 (91.5)	5 (94.4)	5 (94.7)	5 (95.5)	5 (94.3)	5 (93.9)
**Pregnancy type C (%)**	7 (62.1)	7 (90.5)	7 (85.4)	7 (63.0)	7 (89.2)	7 (85.4)
**Robson group C (%)**	11 (22.4)	11 (20.1)	10 (23.8)	10 (80.5)	11 (27.7)	11 (24.4)
Rupture amniotic pocket before delivery C (%)	2 (91.1)	2 (93.6)	2 (89.3)	2 (91.6)	2 (84.6)	2 (88.5)
Smoker C (%)	2 (84.4)	2 (85.2)	2 (87.2)	2 (89.7)	2 (87.9)	2 (88.1)
**Spontaneous delivery C (%)**	2 (70.3)	2 (74.7)	2 (64.8)	2 (64.3)	2 (59.7)	2 (64.9)
**Weeks on admission C (%)**	38.1 (3.5)	38.8 (2.2)	38.9 (1.6)	38.8 (2.4)	38.6 (2.1)	38.7 (2.2)
**Weeks on delivery** $\mu (\sigma )$	38.5 (2.8)	38.9 (2.0)	39.1 (1.7)	39.0 (2.3)	38.9 (2.0)	38.9 (2.0)
Weight on admission $\mu (\sigma )$	81.4 (14.9)	79.5 (14.5)	78.0 (15.2)	79.6 (16.3)	78.3 (14.2)	78.8 (14.5)
**Weight start of pregnancy ** $\mu (\sigma )$	66.4 (14.4)	66.1 (13.5)	65.5 (14.1)	65.5 (14.1)	65.5 (14.4)	66.0 (14.1)

Each hospital is considered a silo. Categorical columns have the number of categories (C) and the percentage of the most frequent (%). Continuous variables have a mean ($\mu$) and standard deviation ($\sigma$). The first row is the number of patients. Bold rows were used as target (n = 19).

**Table 2:** Silos overview part 2.

Variable	Silo 6	Silo 7	Silo 8	Silo 9	Total
N (total)	12002	8258	6693	11786	80874
**Actual type of delivery C (%)**	10 (63.8)	0 (100)	10 (50.1)	9 (64.6)	11 (52.9)
Bishop Score C (%)	14 (99.3)	15 (97.9)	14 (99.2)	15 (95.0)	16 (95.3)
**Blood group C (%)**	13 (41.6)	10 (39.2)	10 (40.1)	10 (41.7)	14 (40.4)
**Body mass index** $\mu (\sigma )$	24.9 (5.1)	24.9 (7.0)	24.8 (8.0)	25.7 (5.6)	25.1 (7.0)
Cervical Consistency C (%)	4 (99.5)	4 (99.7)	4 (99.5)	4 (96.9)	4 (96.5)
Cervical Position C (%)	4 (99.5)	4 (99.7)	4 (99.5)	4 (96.9)	4 (96.5)
**Delivery type C (%)**	6 (54.3)	5 (52.1)	5 (47.8)	5 (59.0)	8 (51.3)
Dilatation C (%)	5 (99.5)	5 (99.7)	5 (99.5)	5 (96.9)	5 (96.5)
Effacement C (%)	5 (99.5)	5 (99.7)	5 (99.5)	5 (96.9)	5 (96.5)
Fetal station C (%)	5 (99.5)	5 (99.7)	5 (99.5)	5 (96.9)	5 (96.6)
**Followed physician C (%)**	3 (96.9)	3 (99.4)	3 (97.8)	3 (99.2)	4 (97.8)
**Followed physician hospital delivery C (%)**	2 (62.1)	2 (63.2)	2 (69.4)	2 (83.1)	2 (69.0)
**Followed physician primary care C (%)**	2 (53.1)	2 (86.7)	2 (63.1)	2 (87.3)	2 (67.6)
Followed physician private clinic C (%)	2 (68.2)	2 (73.5)	2 (71.0)	2 (78.1)	2 (75.8)
Gestational diabetes C (%)	2 (92.2)	2 (88.2)	2 (89.9)	2 (86.8)	2 (89.5)
Induced delivery C (%)	2 (91.9)	2 (85.9)	2 (87.4)	2 (93.9)	2 (92.5)
**Mother age ** $\mu (\sigma )$	31.3 (5.2)	31.4 (5.4)	31.5 (5.6)	30.1 (5.6)	31.1 (5.6)
Nr deliveries forceps C (%)	4 (82.0)	4 (86.0)	3 (94.0)	4 (89.3)	5 (82.6)
Nr deliveries no assistance C (%)	8 (58.8)	9 (61.2)	10 (68.9)	9 (61.5)	12 (60.3)
Nr deliveries vacuum C (%)	4 (78.9)	4 (81.6)	4 (88.0)	5 (82.3)	5 (77.4)
Nr of C-sections C (%)	6 (69.1)	6 (74.5)	5 (85.5)	6 (77.8)	6 (74.6)
**Nr of pregnancies C (%)**	13 (44.2)	9 (42.9)	11 (42.0)	13 (40.2)	19 (42.1)
**Nr of born babies C (%)**	9 (38.4)	9 (42.6)	10 (41.2)	10 (43.2)	12 (38.8)
**Nr of consultations** $\mu (\sigma )$	6.8 (4.0)	7.7 (3.2)	9.3 (4.5)	8.9 (5.5)	8.4 (5.1)
Pelvis Adequacy C (%)	4 (89.6)	4 (52.9)	3 (93.1)	4 (81.5)	4 (82.6)
**Position admission C (%)**	6 (84.5)	7 (61.3)	5 (89.2)	4 (74.2)	7 (73.1)
**Position on delivery C (%)**	5 (93.0)	5 (93.6)	5 (94.8)	5 (94.2)	5 (93.9)
**Pregnancy type C (%)**	7 (88.0)	7 (85.4)	7 (86.0)	7 (92.9)	7 (85.4)
**Robson group C (%)**	11 (27.2)	11 (24.7)	11 (21.4)	11 (26.7)	11 (24.4)
Rupture amniotic pocket before delivery C (%)	2 (85.0)	2 (84.4)	2 (89.9)	2 (93.8)	2 (88.5)
Smoker C (%)	2 (91.0)	2 (90.7)	2 (85.5)	2 (89.9)	2 (88.1)
**Spontaneous delivery C (%)**	2 (64.9)	2 (64.0)	2 (64.7)	2 (62.9)	2 (64.9)
Weeks on admission $\mu (\sigma )$	38.7 (1.8)	39.0 (2.0)	38.6 (2.1)	38.8 (1.9)	38.7 (2.2)
**Weeks on delivery** $\mu (\sigma )$	38.8 (1.8)	39.2 (1.7)	38.7 (2.0)	39.0 (1.6)	38.9 (2.0)
**Weeks on admission** $\mu (\sigma )$	77.7 (13.4)	79.2 (14.7)	76.7 (13.0)	83.1 (15.2)	78.8 (14.5)
**Weight start of pregnancy** $\mu (\sigma )$	65.6 (13.5)	66.0 (13.7)	65.6 (14.1)	67.4 (14.6)	66.0 (14.1)

Each hospital is considered a silo. Categorical columns have the number of categories (C) and the percentage of the most frequent (%). Continuous variables have a mean ($\mu$) and standard deviation ($\sigma$). Abbreviation meaning in the “Appendix”. The first row is the number of patients. Bold rows were used as target (n = 19).

## Methods

The section will cover the steps we took for evaluating the models. We first addressed the preprocessing of the data, then the training of the models and finally the evaluation of the models. The evaluation was done by comparing the performance of the distributed model with the local and centralised models. The performance was measured by the AUROC, AUPRC, RMSE and MAE. The results were then compared using a 2-sample T-test.

### Preprocessing

The initial dataset underwent preprocessing by eliminating attributes that were missing more than 90% of their data across all storage units (or silo). We standardized the representation of missing values, which varied widely, including representations such as “-1” “missing” or simply blank spaces. For imputation, we utilized the mean for continuous variables (calculated within site) and introduced a special category (NULLIMP) for categorical variables. We converted all categories into numerical values based on a predefined mapping that covered all potential categories across the datasets. Although this approach introduces an ordinal relationship and potential bias is created among features, we disregarded this concern because the methodology was uniformly applied across all datasets intended for training local, distributed and centralised. These preprocessing tasks were executed once for each dataset and silo.

However, in the context of training classification models, it is crucial that all classes of the target variable are known at the time of training and are represented in each split of the cross-validation process. To address this, we employed SMOTE^33^ to up-sampled low-frequency target classes. We established a threshold of n<25 for low-frequency variables to ensure that each cross-validation split contained at least two instances of the class-although a minimum of 10 instances (10 splits) might suffice, we opted for 25 to mitigate potential distribution issues and have at least two examples of the class in each split. Additionally, we created dummy rows for missing target classes by imputing the mean for continuous variables and the mode for categorical variables (calculated within site). The necessity for up-sampling and missing variable creation was evaluated and applied as needed for each training session and for each target, considering that each session’s split could result in a training set lacking instances of low-frequency classes.

All procedures were coded in python 3.9.7 with the usage of the scikit-learn library^34^ and mlxtend library^35^.

### Model training

To avoid pitfalls of inductive bias from a certain learning strategy, we learned six different models (i) Decision Trees, (ii) Bayesian methods, (iii) a logistic regression model with Stochastic Gradient Descent, (iv) K-nearest neighbours, (v) AdaBoost and (vi) Multi-layer Perceptron. The decision was to create diversity in the models used, in order to assess if the training methodology could have an impact on distributed model creation. The distributed model was an ensemble of models from each silo on a weighted soft-voting basis. The weights were defined by weighted averages of the scores each model obtained in the training set. Then the final result is obtained by creating a weighted average of the class predictions for classification and a weighted average for regression. A model like this can be implemented with peer-to-peer or federated approaches. Nineteen features were used as target outcomes. These features were selected by filtering by the percentage of null values (below 50%). This choice was related to maintaining a equilibrium between having a wide range of variables to test how the target variables affects the outcome and having target variables that did go through an harsh imputation mechanism. For categorical outcomes, thirteen were selected (AA—Baby’s Position on Admission (like vertex, cephalic or transverse); ANP—Baby’s Position on Delivery (like vertex, cephalic or transverse); AGESTA—Number of Pregnancies; APARA—Number of born babies; GS—Blood Group; GR—Robson Group, which is a system used to categorize all women giving birth into ten groups based on characteristics that are clinically relevant to the outcome of delivery; TG—Pregnancy Type (like spontaneous or In vitro fertilisation); TP—Delivery Type (like vaginal or C-section); TPEE—if the delivery was spontaneous, meaning that no induction was needed; TPNP—Actual Type of Delivery, or the actual delivery method; V—if the mother was followed by physician; VCS—if the mother was followed by a physician in primary care; VNH—if the mother was followed by a physician in the same hospital of the delivery;). For continuous variables, six were selected (IA—Mother’s Age; IGA—Weeks on Admission; IMC—Body Mass Index ; NRCPN—Number of consultations; PI—Weight of the mother at the start of pregnancy; SGP—number of weeks on Delivery). Given the wide range of different variables, there is the potential of using the predictions of the models in the whole pregnancy process. More information about the variables can be seen in Tables 1 and 2. Local models were built with each silo’s data. The centralised model was trained with a training dataset from all the silos combined.

### Model performance evaluation

All models were built for a certain outcome variable with a repeated cross-validation (2 times and 10 splits each) and then compared, over ten stochastic runs, with evaluation being performed on a test set held out from each silo. By performing cross-validation twice, we aimed to generate a more robust estimation of the model’s performance metrics by averaging the results over two separate runs, each partitioning the data differently. This approach is particularly useful in scenarios where data is limited or highly variable, as it provides a clearer insight into the model’s expected performance in unseen data scenarios. The metrics used for classification models were Weighted Area Under the Receiver Operating Characteristic Curve (AUROC) computed as One-versus-Rest, Weighted Area Under the Precision-Recall Curve (AUPRC). The metrics for regression models were Root Mean Squared Error (RMSE) and Mean Absolute Error (MAE). The algorithm is shown in the Algorithm 1. This rendered over 1000 different combinations. When a variable was used as outcome to predict, all others were used as predictors.

**Algorithm 1:**
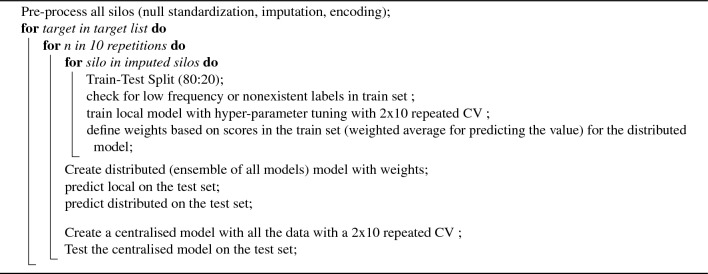
Creation and evaluation of the three different models. We first preprocessed data. Then for each target, we created a distributed and centralised model. Then, over ten repetitions per silo, we created a new train and test set and local model and tested the centralised, distributed and local on this test set.

After all the data was collected, we used the standard independent 2-sample T-test to check if the differences were significant with a $\alpha$ of 0.05. First, we compared the overall performance of the distributed model vs their centralised and local counterpart. We also compared every distributed model per algorithm and sequentially the centralised and correspondent local model across all algorithms and repetitions and outcome variables with 2-sample T-test as well.

## Results

Table 3 shows the aggregated metrics for AUROC, AUPRC, RMSE and MAE for distributed, centralised and local models predicting capabilities on each silo. The data refers to the mean of the metric values for all columns tested as targets for all methods and all silos. We also calculated the 95% confidence interval for each model (local and distributed per silo) in order to assess how well the distributed model would work as opposed to the local one per silo. We also calculated the p Value for the means of the distributed vs centralised and distributed vs local.

Table 4 shows all iterations of the tests and how the distributed model compared with the centralised and the local for each silo, target variable, repetition and machine learning model like described in Algorithm 1. The rows describe the relationship of the distributed and the centralized and the columns the relationship between the distributed model and the corresponding local model.

**Table 3:** Comparison of the distributed model with the centralised model and with the local model (mean for all model and all columns).

		M	SD	95% CI	*P*
AUPRC	distributed	0.691	0.216	(0.686, 0.696)	–
	Centralised	0.706	0.225	(0.701, 0.711)	**1.10e**−**17**
	Local	0.659	0.220	(0.654, 0.665)	**4.71e**−**05**
AUROC	Distributed	0.723	0.182	(0.718, 0.727)	–
	Centralised	0.729	0.180	(0.725, 0.734)	**2.98e**−**26**
	Local	0.692	0.164	(0.688, 0.695)	**2.48e**−**02**
MAE	Distributed	2.370	1.608	(2.315, 2.425)	–
	Centralised	2.365	1.923	(2.298, 2.431)	**2.23e**−**04**
	Local	2.527	1.799	(2.465, 2.589)	9.01e−01
RMSE	Distributed	21.171	46.078	(19.584, 22.757)	–
	Centralised	19.839	28.645	(18.853, 20.826)	**2.92e**−**02**
	Local	23.771	49.776	(22.057, 25.485)	1.63e−01

2-sample T-test for the means was used as hypothesis test. Bold for *P* value below 0.05. AUPRC and AUROC for categorical target variable and RMSE and MAE for continuous target variable.

**Figure 1:**
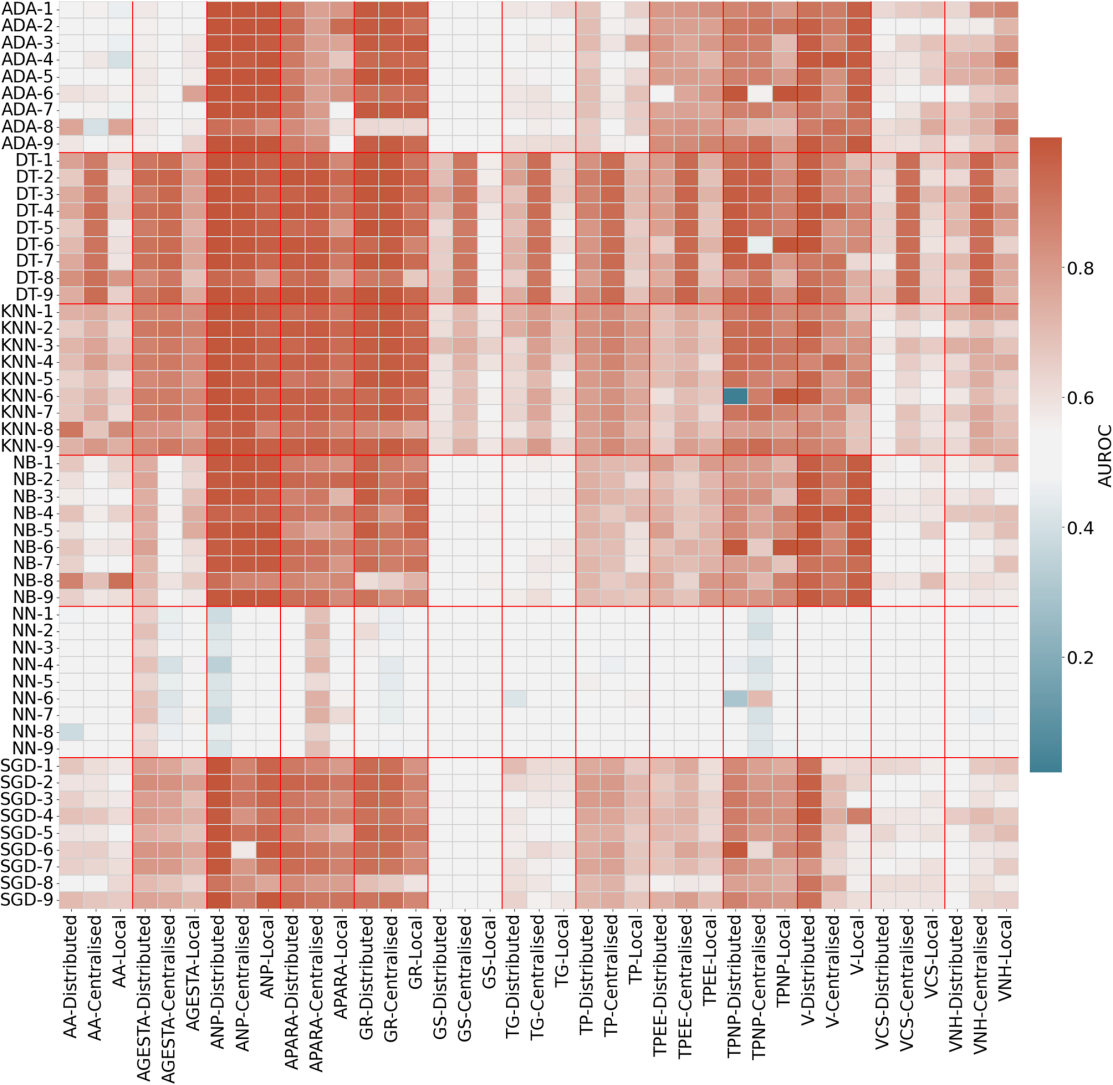
Heatmap of classification algorithm and silo vs target variable and model type. Value is the AUROC mean of all 10 experiments. Y axis is the algorithm and silo. X axis is target variable and method. *AA* position admission, *ANP* position on delivery, *AGESTA* nr of pregnancies, *APARA* nr of born babies, *GS* blood group, *GR* Robson group, *TG* pregnancy type, *TP* delivery type, *TPEE* spontaneous delivery, *TPNP* actual type of delivery, *V* followed physician, *VCS* followed physician primary care, *VNH* followed physician hospital delivery.

**Figure 2:**
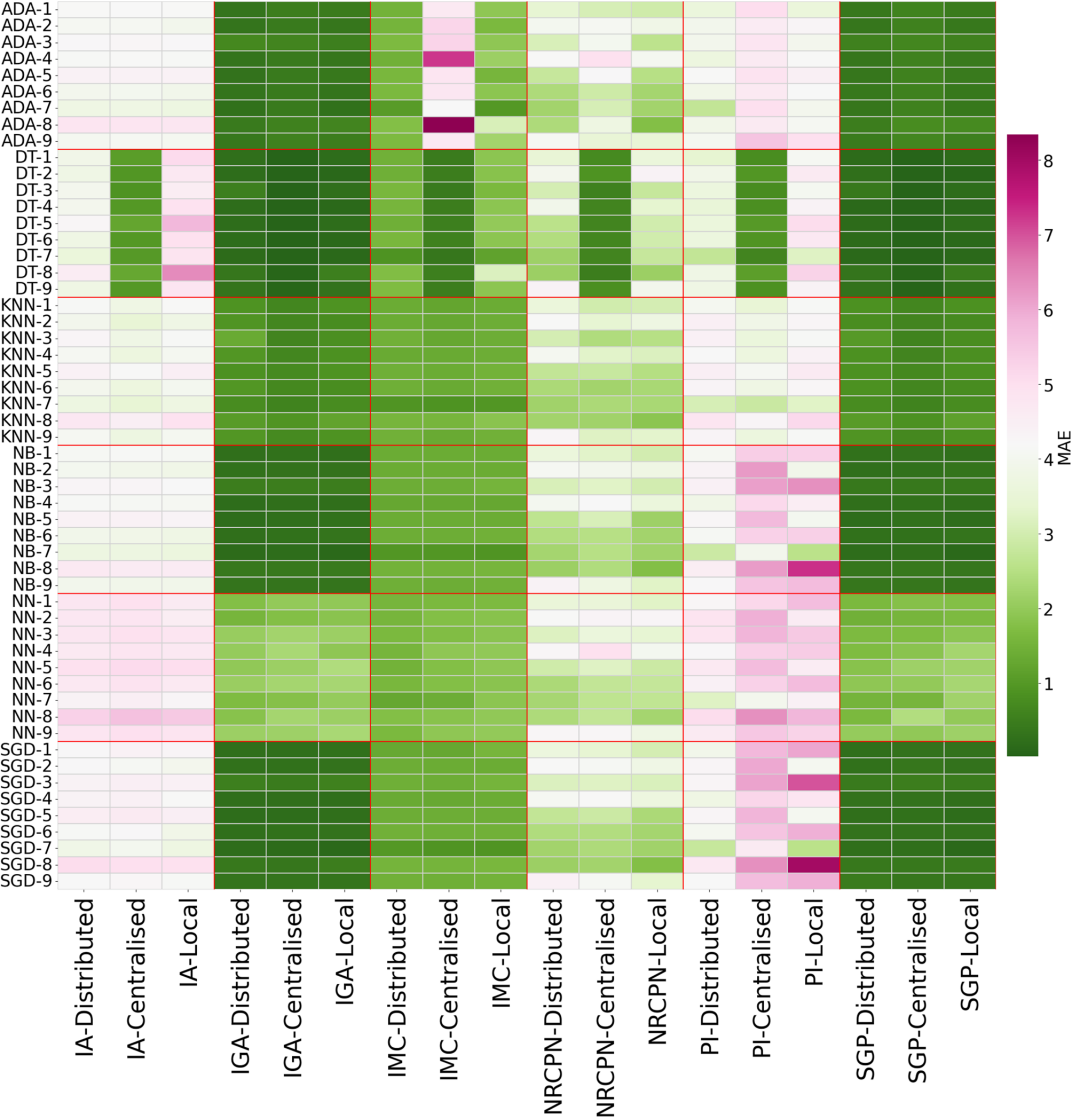
Heatmap of regression algorithm and silo vs target variable and model type. Value is the MAE mean of all 10 experiments. The y axis is the algorithm and silo. X axis is target variable and method. *IA* mother age, *IGA* weeks on admission, *IMC* BMI, *NRCPN* nr of consultations, *PI* weight start of pregnancy, *SGP* weeks on delivery.

Figure 1 shows the AUROC of each algorithm and silo on the Y axis and target variable and type of model on the X. The color bar refers to the value of the AUROC. Blue being lower values and red bigger values. The same type of graph was created for regression, where the Fig. 2 shows the MAE for each silo and algorithm and target variable and type of model.

**Table 4:** Model comparison: distributed versus centralised and local for every test.

		Distributed > local	Distributed = local	Distributed < local	Row total
SGD	Distributed > centralised	72 (7.0)	14 (1.4)	9 (0.8)	95 (9.3)
	Distributed = centralised	14 (1.4)	17 (1.7)	6 (0.6)	37 (3.6)
	Distributed < centralised	11 (1.1)	11 (1.1)	17 (1.7)	39 (3.8)
NN	Distributed > centralised	44 (4.3)	44 (4.3)	7 (0.7)	95 (9.3)
	Distributed = centralised	2 (0.2)	33 (3.2)	2 (0.2)	37 (3.6)
	Distributed < centralised	0 (0)	17 (1.7)	22 (2.1)	39 (3.8)
KNN	Distributed > centralised	16 (1.6)	0 (0)	1 (0.1)	17 (1.7)
	Distributed = centralised	10 (1)	2 (0.2)	1 (0.1)	13 (1.3)
	Distributed < centralised	72 (7)	28 (2.7)	41 (4)	141 (13.7)
ADA	Distributed > centralised	64 (6.2)	25 (2.4)	22 (2.1)	111 (10.8)
	Distributed = centralised	5 (0.5)	12 (1.2)	10 (1)	27(2.6)
	Distributed < centralised	10 (1)	6 (0.6)	17 (1.7)	33 (3.2)
NB	Distributed > centralised	51 (5)	19 (1.9)	34 (3.3)	104 (10.1)
	Distributed = centralised	5 (0.5)	19 (1.9)	12 (1.2)	36 (3.5)
	Distributed < centralised	3 (0.3)	4 (0.4)	24 (2.3)	31 (3)
DT	Distributed > centralised	27 (2.6)	0 (0)	1 (0.1)	28 (2.7)
	Distributed = centralised	8 (0.8)	0 (0)	0 (0)	8 (0.8)
	Distributed < centralised	97 (9.5)	12 (1.2)	26 (2.5)	135 (13.2)
Total		511 (49.8)	263 (25.6)	252 (24.6)	1026 (100)

Each cell is the total of distributed model when compared with centralised model (row) and local model (column) across different silos and outcome variable. (> for better, = for non significance and < for worse). The first example is 72 which means that 72 iterations of the distributed SGD was better than the centralised and local. Comparison was done with 2-sample T-test with a $\alpha$ of 0.05 (% in parenthesis).

*SGD* stochastic gradient descent, *NN* neural network, *KNN* K-nearest neighbors, *ADA* AdaBoost, *NB* naive Bayes, *DT* decision tree.

## Discussion

A significant finding is that nearly 59% of distributed models demonstrated comparable, if not superior, performance relative to their centralized counterparts (Table 4 last column, first two values for each algorithm). From these, 41.9% were also better or equal to the local model. Using the best-performing algorithm (SGD), we observe a 77.2% improvement in distributed settings compared to centralized, and a 66% improvement over both centralized and local models. This outcome underlines the potential of distributed models to offer reliable inference capabilities that match those of traditional centralized models, without sacrificing predictive accuracy. Furthermore, the adoption of distributed models enhances privacy for data owners, presenting a compelling case for their broader application in data-sensitive environments. Overall, our results suggest that it is possible to implement a distributed model without significantly losing information. Our analysis suggests that SGD, Adaboost and Naive Bayes approaches are suitable for such distributed approached with tabular data. In contrast, MLPerceptron, Decision Trees and KNN do not seem to be a good approach for such use cases.

However, there are still issues to be addressed. This methodology presents hurdles regarding categorical class handling. Firstly, all classes should be known first-hand and should be given to each model even if that silo in particular has no cases of that class. Secondly, low-frequency classes are also an issue to be addressed, since training the model with cross-validation will raise problems because each split should have all classes present. Our approach relied on sample creation for low and non-existent target classes. However, this approach is adding information to the model that is not originally there. The way we chose for minimising this issue was by creating dummy variables with median and mode imputations based only on the information in the dataset. Nevertheless, non-existent classes are impossible to address without prior information. These class problems could be partially tackled in production by implementing data management and governance procedures, namely data dictionaries. Still on data preprocessing, we applied ordinal encoding to the variables which will create a natural hierarchy between variables. One solution for this is to create binary columns for each class in each column. This will remove the hierarchy between classes but increase variable numbers and training time considerably.

Another issue to consider is the path adopted to build the distributed model. In this case, it was decided to develop an ensemble of models with voting. However, other methods could have been employed, like parameter averaging, that should be tested as well. In particular, the usage of more robust neural networks could be assessed as well. We chose not to test state-of-the-art neural networks since the data volume was low for that use case and several papers have already demonstrated that neural networks are not the most suitable tool for tabular data^36,37^. We chose to add MLPerceptron as a baseline for comparison with the remaining algorithms. The results show us that the performance was below the other algorithms, but in this concrete case, the problem may reside in the architecture chosen and hyperparameters used in the Cross-validation which may have lead to underfitting. Despite this, a precise and thorough demonstration of this use case would be important to consider such scenarios.

Furthermore, the algorithm underlying the distributed model is of importance as well for its performance versus the centralised model. Figs. 1 and 2 and Table 4 show us that Decision trees and K-nearest neighbours implemented in a centralised manner are consistently better than the distributed counterpart. This is specially notorious in the case of the decision trees. We believe this may be related to way the algorithm is implemented. A centralised version may be able to create optimal splits in the data, while the distributed version may not be able to do so. This is a topic that should be further explored.

Even though this improvement may have a relationship to the target variable (i.e. Fig. 2 for IA and IGA variables), it is still an important fact to take into account when implementing such architectures. The performance of the models is also interesting to catch differences in silos. See silo 6 for TPNP (Fig. 1) where silo 6 consistently behaves differently than the rest. Checking performance data regarding regression tasks, we can see a drop in performance for PI and IA. While the explanation for the performance of IA can be explained by the average value of it which is 66, which is the highest average in the dataset. This means that the model will have a harder time predicting these values, being also true for the distributed model. This is a topic that should be further explored.

As for implementation, specially preprocessing, we found that having the description of data across silos is vital since we might need to convert and encode data. This is a step that should be taken into account when implementing such a system. This might be easier to implement in a federated manner, where a central orchestrator could take care of this. If an implementation like peer-to-peer is implemented, the metadata should also be shared or defined a priori. Other important issue is related with absence of data and missing values and categories. Most machine learning models expect a specific size of input data. This is a problem when we have missing values or categories that are not present in the training set. Our approach was to handle it with synthetic data generation which may suffice for most scenarios. Regarding the prediction capability as a whole, we found that this data is suitable to apply machine-learning models in order to predict several clinical outcomes, with very good results for several target variables.

## Conclusion

This study represents a comprehensive evaluation of distributed machine learning using real-world tabular obstetrics data from nine distinct sources on such a significant scale. It encompasses a variety of algorithms and outcome variables, comparing these to both centralized and local approaches. Our work demonstrated the performance of distributed models using real-world data by comparing their performance with that of local models, which are trained with data from individual silos, and centralized models, which utilize data from all silos. The findings reveal that an ensemble of models, essentially a distributed model as investigated in this study, can capture the nuances of the data, achieving performance comparable to a model constructed with comprehensive data. Although the performance of these models is shaped by factors such as the inherent characteristics of the target variables and the data distribution across different silos, we are now fairly confident that distributed learning represents a significant advancement. Particularly, if a distributed model can match or surpass the performance of a centralized model, this is notably beneficial. Such an outcome underscores the value of distributed models as they not only maintain, but potentially enhance, predictive accuracy while offering a higher degree of data privacy compared to centralized systems. This balance of privacy with efficiency is especially crucial in fields where data sensitivity is paramount, making distributed learning an appealing option when evaluated against both centralized and local models. Considering the robust performance metrics observed, with AUROC/AUPRC scores exceeding 80% and MAE maintained below 1, further investigation into distributed models is warranted. Specifically, we aim to develop distributed models for predicting clinical outcomes, such as delivery type or Robson Group classifications, which hold significant potential for real-world clinical application like reducing unnecessary Cesarean Sections or accelerating diagnosis. Our findings highlight that distributed learning not only advances data privacy while maintains high prediction accuracy, promising substantial benefits for clinical practices.

## Data Availability

The data that support the findings of this study are available from the source hospitals but restrictions apply to the availability of these data, which were used under license for the current study, and so are not publicly available. Data are however available from the authors upon reasonable request and with permission of the hospitals ethics committee and privacy officers. The code used to generate the results and graphics is available here: https://github.com/joofio/Evaluating-distributed-learning-algorithms-on-real-world-healthcare-data.

## A: Data dictionary

**Table:**

Initial	Description
IA	Mother age
GS	Blood group
PI	Weight at the beginning of pregnancy
PAI	Weight on admission
IMC	BMI
CIG	If smoker during pregnancy
APARA	Number of previously born babies
AGESTA	Number of pregnancies
EA	Number of previous eutocic deliveries with no assistance
VA	Number of previous eutocic deliveries with help of vacuum extraction
FA	Number of previous eutocic deliveries with help of forceps
CA	Number of previous C-sections
TG	Pregnancy type (spontaneous, in vitro fertilisation...)
V	If the pregnancy was accompanied by physician
NRCPN	Number of prenatal consultations
VH	If the pregnancy was accompanied by a physician in a hospital
VP	If the pregnancy was accompanied by a physician in a private clinic
VCS	If the pregnancy was accompanied by a physician in a primary care facility
VNH	If the pregnancy was accompanied by a physician in the hospital the delivery was made
B	Pelvis adequacy
AA	Baby’s position on admission
BS	Bishop score
BC	Bishop score cervical consistency
BDE	Bishop score fetal station
BDI	Bishop score dilatation
BE	Bishop score effacement
BP	Bishop score cervical position
IGA	Number of weeks on admission
TPEE	If the delivery was spontaneous
TPEI	If the delivery was induced
RPM	If there was a rupture of the amniotic pocket before delivery began
DG	Gestational diabetes
TP	Delivery type
ANP	Baby’s position on delivery
TPNP	Actual type of delivery
SGP	Pregnancy weeks on delivery
GR	Robson group

## References

[1] Ravì D (2017). Deep learning for health informatics. IEEE J. Biomed. Health Inform..

[2] Char, D. S., Shah, N. H. & Magnus, D. Implementing machine learning in health care—Addressing ethical challenges. *N. Engl. J. Med.* **378**, 981–983 (2018). 10.1056/NEJMp1714229 PMC5962261 29539284

[3] Albrecht, J. P. How the GDPR will change the world. *Eur. Data Protect. Law Rev. ***2**, 287–289. https://web.archive.org/web/20211014090922. https://edpl.lexxion.eu/article/EDPL/2016/3/4 (Lexxion Publisher, 2016).

[4] Office for Civil Rights. *Guidance Regarding Methods for De-identification of Protected Health Information in Accordance with the Health Insurance Portability and Accountability Act (HIPAA) Privacy Rule*. (U.S. Department of Health and Human Services, 2013).

[5] Abdulrahman, S. *et al.* A survey on federated learning: The journey from centralized to distributed on-site learning and beyond. *IEEE Internet Things J.* (2021).

[6] Warnat-Herresthal S (2021). Swarm learning for decentralized and confidential clinical machine learning. Nature.

[7] Rajkomar, A., Dean, J. & Kohane, I. Machine learning in medicine. *N. Engl. J. Med.* (2019). 10.1056/NEJMra1814259 30943338

[8] Xu, J. *et al.* Federated learning for healthcare informatics. *J. Healthc. Inform. Res. *. arXiv:3320.4939 (2020). 10.1007/s41666-020-00082-4 PMC7659898 33204939

[9] Yang YC (2021). Influential usage of big data and artificial intelligence in healthcare. Comput. Math. Methods Med..

[10] Wang F, Preininger A (2019). AI in health: State of the art, challenges, and future directions. Yearb. Med. Inform..

[11] Jatain, D., Singh, V. & Dahiya, N. A contemplative perspective on federated machine learning: Taxonomy, threats & vulnerability assessment and challenges. *J. King Saud Univ. Comput. Inf. Sci.* (2021).

[12] Tuladhar, A., Gill, S., Ismail, Z. & Forkert, N. D. Building machine learning models without sharing patient data: A simulation-based analysis of distributed learning by ensembling. *J. Biomed. Inform.* **106**, 103424. https://web.archive.org/web/20210625175422. https://www.sciencedirect.com/science/article/pii/S1532046420300526 (2020). 10.1016/j.jbi.2020.103424 32335226

[13] Xu J (2021). Federated learning for healthcare informatics. J. Healthc. Inform. Res..

[14] Lee GH, Shin S-Y (2020). Federated learning on clinical benchmark data: Performance assessment. J. Med. Internet Res..

[15] Prayitno (2021). A systematic review of federated learning in the healthcare area: From the perspective of data properties and applications. Appl. Sci..

[16] Álvarez Sánchez R, Beristain Iraola A, Epelde Unanue G, Carlin P (2019). TAQIH, a tool for tabular data quality assessment and improvement in the context of health data. Comput. Methods Prog. Biomed..

[17] Di Martino, F. & Delmastro, F. Explainable AI for clinical and remote health applications: A survey on tabular and time series data. *Artif. Intell. Rev.* 1–55. 10.1007/s10462-022-10304-3. arXiv:3632.0613 (2022). 10.1007/s10462-022-10304-3 PMC9607788 36320613

[18] Payrovnaziri SN (2020). Explainable artificial intelligence models using real-world electronic health record data: A systematic scoping review. J. Am. Med. Inform. Assoc. JAMIA.

[19] McElfresh, D. *et al.* When Do Neural Nets Outperform Boosted Trees on Tabular Data? arXiv:2305.02997 (2023).

[20] Klambauer, G., Unterthiner, T., Mayr, A. & Hochreiter, S. *Self-Normalizing Neural Networks*. arXiv:1706.02515 (2017).

[21] Borisov, V. *et al.* Deep neural networks and tabular data: A survey. *IEEE Trans. Neural Netw. Learn. Syst.* 1–21 10.1109/TNNLS.2022.3229161. arXiv:2110.01889 (2022). 10.1109/TNNLS.2022.3229161 37015381

[22] Grinsztajn, L., Oyallon, E. & Varoquaux, G. Why do tree-based models still outperform deep learning on tabular data? 10.48550/arXiv.2207.08815. arXiv:2207.08815 (2022).

[23] Peek N, Rodrigues PP (2018). Three controversies in health data science. Int. J. Data Sci. Anal..

[24] Deist TM (2017). Infrastructure and distributed learning methodology for privacy-preserving multi-centric rapid learning health care: euroCAT. Clin. Transl. Radiat. Oncol..

[25] Price, G., van Herk, M. & Faivre-Finn, C. Data mining in oncology: The ukCAT project and the practicalities of working with routine patient data. *Clinical Oncology (Royal College of Radiologists (Great Britain))* **29**, 814–817, 10.1016/j.clon.2017.07.011 (2017). 10.1016/j.clon.2017.07.011 28781199

[26] Liu D, Fox K, Weber G, Miller T (2022). Confederated learning in healthcare: Training machine learning models using disconnected data separated by individual, data type and identity for Large-Scale health system Intelligence. J. Biomed. Inform..

[27] Kirienko M (2021). Distributed learning: A reliable privacy-preserving strategy to change multicenter collaborations using AI. Eur. J. Nucl. Med. Mol. Imaging.

[28] Wang Y (2019). A fast divide-and-conquer sparse Cox regression. Biostatistics (Oxford, England).

[29] Chandiramani K, Garg D, Maheswari N (2019). Performance analysis of distributed and federated learning models on private data. Proc. Comput. Sci..

[30] Lee GH, Shin S-Y (2020). Federated learning on clinical benchmark data: Performance assessment. J. Med. Internet Res..

[31] Li S (2023). Federated and distributed learning applications for electronic health records and structured medical data: A scoping review. J. Am. Med. Inform. Assoc..

[32] VirtualCare. *Obscare.* https://virtualcare.pt/portfolio/vc-obscare-2-2/. Accessed 26 Feb 2024 (2024).

[33] Chawla NV, Bowyer KW, Hall LO, Kegelmeyer WP (2002). SMOTE: Synthetic minority over-sampling technique. J. Artif. Intell. Res..

[34] Pedregosa F (2011). Scikit-learn: Machine learning in Python. J. Mach. Learn. Res..

[35] Raschka, S. Mlxtend: Providing machine learning and data science utilities and extensions to Python’s scientific computing stack. *J. Open Source Softw.*10.21105/joss.00638 (2018).

[36] Grinsztajn, L., Oyallon, E. & Varoquaux, G. Why do tree-based models still outperform deep learning on tabular data? arXiv:2207.08815 (2022).

[37] Borisov, V. *et al.**Deep Neural Networks and Tabular Data: A Survey*10.48550/arXiv.2110.01889 (2022).

